# Data-Driven Maturity Level Evaluation for Cardiomyocytes Derived from Human Pluripotent Stem Cells (Invited Paper)

**DOI:** 10.3390/electronics13244985

**Published:** 2024-12-18

**Authors:** Yan Hong, Xueqing Huang, Fang Li, Siqi Huang, Qibiao Weng, Diego Fraidenraich, Ioana Voiculescu

**Affiliations:** 1 Department of Computer Science, New York Institute of Technology, Old Westbury, NY 11568, USA; 2 Department of Mechanical Engineering, New York Institute of Technology, Old Westbury, NY 11568, USA; 3 School of AI and Advanced Computing, Xi’an Jiaotong-Liverpool University, Suzhou 215123, China; 4 Department of Cell Biology & Molecular Medicine, Rutgers New Jersey Medical School, Newark, NJ 07101, USA; 5 Department of Mechanical Engineering, City College of New York, New York, NY 10031, USA

**Keywords:** cardiovascular diseases, hPSC-CM maturity, gene expression, cardiac gene selection, culture time prediction

## Abstract

Cardiovascular disease is a leading cause of death worldwide. The differentiation of human pluripotent stem cells (hPSCs) into functional cardiomyocytes offers significant potential for disease modeling and cell-based cardiac therapies. However, hPSC-derived cardiomyocytes (hPSC-CMs) remain largely immature, limiting their experimental and clinical applications. A critical challenge in current in vitro culture systems is the absence of standardized metrics to quantify maturity. This study presents a data-driven pipeline to quantify hPSC-CM maturity using gene expression data across various stages of cardiac development. We determined that culture time serves as a feasible proxy for maturity. To improve prediction accuracy, machine learning algorithms were employed to identify heart-related genes whose expression strongly correlates with culture time. Our results reduced the average discrepancy between predicted and observed culture time to 4.461 days and *CASQ2* (Calsequestrin 2), a gene involved in calcium ion storage and transport, was identified as the most critical cardiac gene associated with culture duration. This novel framework for maturity assessment moves beyond traditional qualitative methods, providing deeper insights into hPSC-CM maturation dynamics. It establishes a foundation for developing advanced lab-on-chip devices capable of real-time maturity monitoring and adaptive stimulus selection, paving the way for improved maturation strategies and broader experimental/clinical applications.

## 1. Introduction

Cardiovascular diseases (CVDs) remain the leading cause of death worldwide, according to reports from the American Heart Association and the National Institutes of Health [1]. In 2021, CVDs were responsible for 20.5 million deaths, accounting for one-third of all global fatalities [2]. In the United States, one person dies from cardiovascular disease every 33 s, with heart disease imposing an economic burden of approximately USD 252.2 billion annually from 2019 to 2020 [3]. These statistics highlight the urgent need for effective treatments for heart disease.

Human pluripotent stem cell-derived cardiomyocytes (hPSC-CMs) represent a promising solution by providing a potentially unlimited cell supply for applications such as cell-based cardiac regeneration therapy, drug toxicity screening [4], and cardiovascular disease modeling [5]. However, curing myocardial infarction, the leading cause of death among adults, requires mature hPSC-CMs that closely resemble adult cardiomyocytes (CMs). Immature hPSC-CMs, with their inherently faster beating rates, pose the risk of inducing lethal arrhythmias if transplanted into recovering adult hearts. This emphasizes the critical need not only to differentiate CMs from hPSCs but also to effectively promote their maturation.

To enhance the maturity and functionality of hPSC-CMs, researchers have explored several approaches, such as mechanical stress stimulation, electrical stimulation, biochemical cues, 3D cardiac tissue remodeling, substrate stiffness modification, combinatorial co-culture to harness paracrine effects, and mitochondrial proteomic analysis to support metabolic development [6–8]. Despite these efforts, hPSC-CMs generated in vitro still exhibit characteristics of neonatal (immature) cardiomyocytes, falling short in cardiac markers, action potential profiles, and morphology compared to mature CMs [9,10]. This immaturity significantly limits their experimental and clinical applications.

Since the maturation process of hPSC-CMs is influenced by numerous factors, developing efficient strategies requires a deep understanding of how individual or combined factors drive maturation. Currently, hPSC-CM maturity is assessed using various biological methods that examine aspects such as cell morphology and structure, electrophysiology, calcium handling, and gene expression [11,12]. However, a significant challenge with existing in vitro hPSC-CM culture systems is the lack of standardized metrics and protocols for quantifying overall maturity. This limitation hinders both the comprehensive evaluation of maturation stages and the development of effective strategies to enhance maturation.

Similar to human development, where an individual’s true biological age is unobservable but can be inferred through various proxies (e.g., chronological age, bone age, and telomere length), the maturity level of hPSC-CMs can be approximated using measurable indicators such as post-differentiation time and biological markers (e.g., gene expression profiles). Based on this hypothesis, we propose a data-driven approach to address the challenges associated with assessing hPSC-CM maturity in current in vitro culture systems. A data-driven model is a technology based on the analysis of the data about a specific system that can find the relationship between variables in the system without explicit knowledge of the physical behavior of the system [13–15].

Our proposed approach will first assess the feasibility of approximating true maturity levels using observable metrics. Subsequently, machine learning algorithms will be applied to identify biological measurements that strongly correlate with these maturity proxies, enabling more accurate predictions of maturity levels. This method not only enhances our understanding of the maturation process but also uncovers latent maturation states. In particular, with 48,804 gene probe data at multiple in vitro culture time points [16], the data-driven model is a promising method for the quantitative evaluation of the hPSC-CM maturity level. Based on the gene profile data, which span different stages of cardiac development, we have verified that hPSC-CMs can continually mature toward more adult-like hPSC-CMs up to 120 days. Then, by adopting in vitro culture duration as the proxy to estimate the maturity level of hPSC-CMs, these gene probes were ranked based on their importance to predict the culture duration. Specifically, this ranking of gene importance reflects the weight assigned to each gene in the machine learning-based prediction model, indicating its contribution to explaining the variance in culture time. Five different predictive methods have been proposed and the results showed that the most accurate culture time prediction has an average error of 4.461 days, and the most important cardiac gene that correlates to the culture time is Gene *CASQ2* (Calsequestrin 2), whose protein plays a role in the storage and transportation of calcium ions.

The major features of this work are as follows. (1) The existing hPSC-CM maturity quantification schemes mainly focus on deciding whether the hPSC-CMs are mature or not. For the first time, we have proposed a data-driven pipeline that is capable of quantifying the maturity level of the cell in vitro with a finer granularity. (2) The selected dominating cardiac genes can guide the design of more efficient stimulation schemes to promote hPSC-CM maturation. (3) The data-driven approach opens the door of hPSC-CM study to multidisciplinary researchers without a strong biology background.

The remainder of the paper is organized as follows. Section 2 introduces existing biological studies on the maturity determination methods for hPSC-CMs. Section 3 verifies the feasibility of adopting in vitro culture duration as the proxy to estimate the maturity level of hPSC-CMs. Section 4 explains the cardiac gene selection and culture duration prediction models. Section 5 presents the results, Section 6 discusses the limitations of the study and future work, and Section 7 concludes the paper.

## 2. Related Works

**Figure 1.**
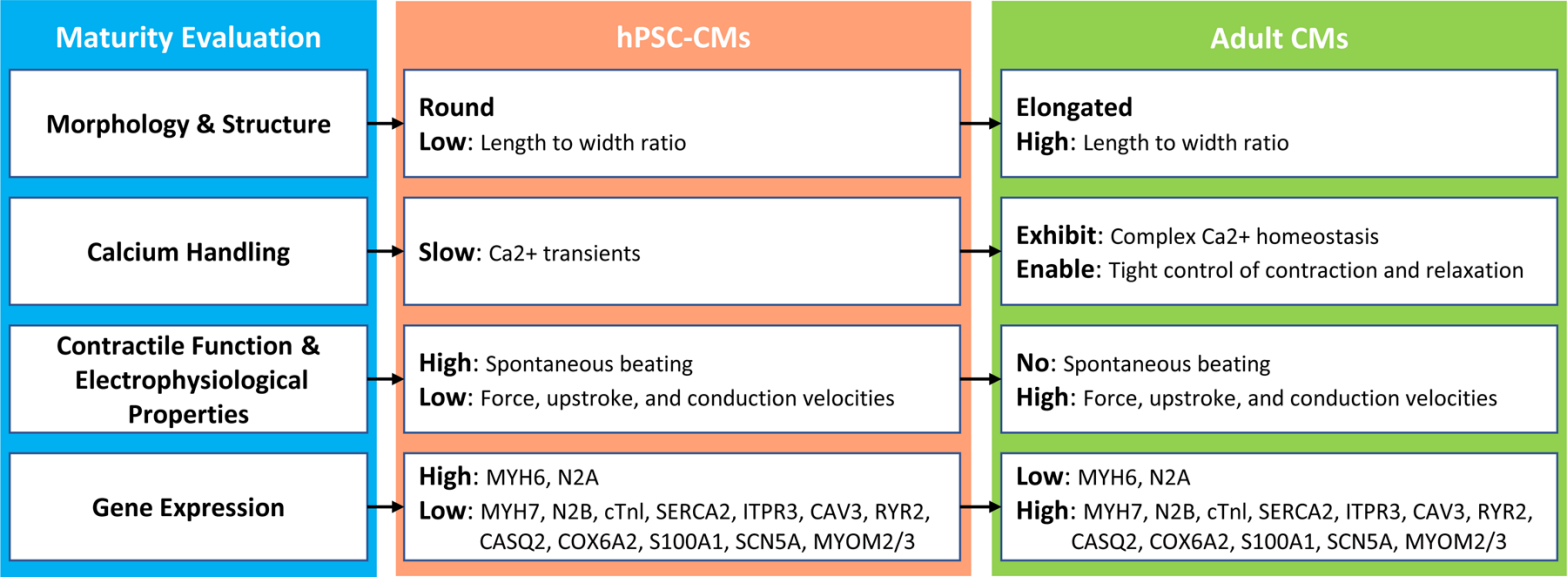
Existing maturity evaluation methods of hPSC-CMs.

As compared to human adult cardiomyocytes, the current in vitro hPSC-CMs remain largely immature. By the principle of “if you cannot measure it, you cannot improve it” [17], the slowly moving hPSC-CM maturation research is challenged by the lack of a convenient and standard method to assess hPSC-CMs’ degree of maturity quantitatively. Currently, researchers have been using various biological methods to evaluate whether hPSC-CMs are becoming more mature towards adult CMs from various aspects: morphology and structure, calcium handling, contractile function and electrophysiological properties, and gene expression. The differences between immature CMs and mature CMs in these four aspects are summarized in Figure 1. The details of these aspects are provided below.

### 2.1. Morphology and Structure

The process from human fetal CMs to adult phenotype in vivo takes 6 to 10 years [18]. However, hPSC-CMs can be generated within 15 days of in vitro differentiation [19]. To nurture more mature hPSC-CMs, researchers increased culturing time up to 120 days and found key changes in cultured hPSC-CMs [11]. The main difference can be classified into four parts: (1) shape of the cell; (2) sarcomeres: a contractile unit of the muscle fiber; (3) sarcoplasmic reticulum (SR): also a structure found within muscles, the main function of which is to store calcium ions Ca^2+^; and (4) transverse tubules (T-tubules): a cell membrane that penetrates the center of the skeletal and cardiac muscle cell.

As illustrated in Figure 1, adult cardiomyocytes are well aligned, rod-like, multinucleated cells. They have highly organized sarcomeres and well-developed SR and T-tubules. In immature hPSC-CMs, those features tend to be lacking. The hPSC-CMs are small, rounded, mononucleated, with disorganized and shorter sarcomeres. Moreover, hPSC-CMs have poorly developed SR and no T-tubules [12].

Data Collection and Processing Methods: During the culturing period of 120 days, the status of hPSC-CMs can be divided into two stages: the early stage (from 20 days to 40 days) and the late stage (from 80 days to 120 days). During the culture process, the hPSC-CMs are observed under the microscope. Cell images are captured and saved into a computer. The parameters of sarcomere length, cell perimeter, cell area, percentage of multinucleation, and circularity index (ratio between the cell width and length) are obtained with appropriate image analysis software [11].

### 2.2. Calcium Handling

In adult CMs, T-tubules and SR are well developed to regulate Ca^2+^ induced Ca release (CICR) and fast excitation-contraction coupling (ECC). The sharp and uniform increase of intracellular Ca^2+^ concentration in adult CMs is important for the synchronized contraction in multiple sarcomeres. By contrast, in hPSC-CMs, T-tubules are absent and SR is underdeveloped with low expression for the most part of sarcoendoplasmic reticulum calcium ATPase (SERCA) and other key proteins.

Data Collection and Processing Methods: Calcium imaging technology refers to a method for monitoring calcium ion concentration in tissues using calcium ion indicators named dyes. The cultured slip is observed under the microscope after placing the coverslip cultured with hPSC-CMs in a Petri dish containing the dye solution for about 30 min at 37 degrees Celsius. Images are captured and quantified with appropriate image analysis software.

### 2.3. Contractile Function and Electrophysiological Properties

The contractile function is a fundamental status indicator of CMs. (1) The hPSC-CMs and fetal CMs display a comparable force generation capacity (0.22 ± 0.70 mN/mm^2^ to 11.8 ± 4.5 mN/mm^2^ in hPSC-CMs and ∼0.4 mN/mm^2^ in fetal CMs), while adult CMs generate much larger forces (∼51 mN/mm^2^) [20]. (2) The hPSC-CMs also show immaturity in their electrophysiological properties, as compared with adult CMs, including reduced electrical excitability, higher resting membrane potential (−20 to −60 mV vs. ∼−90 mV), low capacitance (30–50 pF vs. ∼150 pF), smaller upstroke (15–50 V/s vs. 180–400 V/s) and conduction velocity (2.1–20 cm/s vs. 41–84 cm/s), and presence of spontaneous beating, which is found in early fetal CMs [21].

Data Collection and Processing Methods: Contractile and electrophysiological data are monitored and collected in real time by devices such as HEKA EPC-10 patch-clamp amplifier. The changes in contractile and electrophysiological properties are analyzed by appropriate software, e.g., Patchmaster and Igor Pro [22].

### 2.4. Gene Expression

During the culturing of hPSC-CMs, several cardiac-specific genes express a more adult heart-like expression level over time, such as *CASQ2*, *CRYAB*, *MYH6*, *MYH7*, *TNNI3*, and *ACTC1*. (1) Gene *CASQ2* results in a more mature calcium handling phenotype during culturing [23]. The *CASQ2* gene provides instructions for marking a protein called calsequestrin 2 found in myocytes, where it is involved in storing and transporting calcium ions. (2) *CRYAB* is a part of the small heart protein family and functions as a molecular chaperone that primarily binds misfolded proteins to prevent protein aggregation, inhibit apoptosis, and contribute to in-tricellular architecture. (3) In an adult heart, the genes *MYH6* and *MYH7* are predominantly expre d in the ventricle and atrium, respectively. As the culture time of hPSC-CMs is prolonged, the gene expression level of *MYH7* and *MYH6* tends to mature toward the adult cardiomyocyte level. Also, *MYH7* and *MYH6* provide e ntial instructions to form part of a large protein called type II myosin that generates the mechanical force to pump blood to the rest of the body. (4) The level of *TNNI3* expression gradually increases with the culture time. The *TNNI3* gene provides instruction for making a protein called cardiac troponin I, which helps coordinate the heart’s contraction. (5) *ACTC1* is the major protein of the thin filament in cardiac sarcomeres. Some other important cardiac-specific genes are listed in Table 1.

Data Collection and Processing Methods: The differentiation protocol is performed over millions of cells. At different culture dates, these cells are removed for RNA sample collection, and total RNA from hPSC-CMs can be isolated using reagent by following the manufacturer’s protocol [24,25]. To obtain the gene expression profiles, 48,804 genes are probed with the microarray method, which can determine whether the RNA or DNA from a particular individual contains a gene mutation. The gene expression profile of hPSC-CMs is then compared with adult CMs by conducting statistical tests.

## 3. The hPSC-CM Maturity Evaluation Proxy

To propose a data-driven model capable of quantifying the maturation stage of hPSC-CMs with fine granularity, we first examine the gene expression data collected during culturing.

### 3.1. Cardiac-Specific Gene Data Collection

The miRNA expressions of hPSC-CMs (Supp_table4 in [25]) are adopted as input data to analyze the maturation process. The gene expression datasets include (a) the mRNA expression collected for a set of in vitro culture time points in $T=\left\{0,\;3,\;7,\;10,\;14,\;20,\;28,\;35,\;45,\;60,\;90,\;120\right\}$ days; (b) two independent runs of the adult mRNA expression, namely adult 1 sample and adult 2 sample, where the culture time is indicated as $t^{*}>\;>120$, approximately two years.

On day t∈T, three million cells were sampled for RNA collection and $N=\left\{1,\;\ldots ,\;203\right\}$ cardiac genes listed in the Genomic Institute of the Novartis Research Foundation (GNF) expression atlas were probed [26]. For the i-th probed cardiac gene expression, i∈N, the fold change value $y_{i}^{t}$ is measured, which is used to describe the degree of change from the initial gene profile of the fetal sample to the gene profile on day t.

(1) $$y_{i}^{t}=\log_{2}\left(\frac{z_{i}^{t}}{z_{i}^{t_{0}}}\right),\;\forall i\in N\;\forall t\in T,$$

where for the i-th gene, $z_{i}^{t}$ indicates the corresponding gene probe profile of the hPSC-CMs on day t, and $z_{i}^{t_{0}}$ transformed the ratio The $log_{2}$ is the initial profile of the fetal hPSC-CMs. to “fold”, i.e., “times”, where $y_{i}^{t}=1$ means $z_{i}^{t}$ is doubling $z_{i}^{t_{0}}$.

The resulting fold change dataset given below will be used to verify whether culture time can serve as the proxy of the maturity level of hPSC-CMs.

(1) A set of fold change values ${ϒ}_{t}=\left\{y_{i}^{t}\;|\;i\;\in \;N\right\}$ for hPSC-CMs on day t∈T, where $y_{i}^{t}$ is the fold change value of the i-th gene. (2) ${ϒ}^{*}=\left\{y_{i}^{*}\;|\;i\in N\right\}$ for adult CMs on day $t^{*}$, where $y_{i}^{*}$ is the fold change value of the i-th gene.

### 3.2. Maturation Level vs. Culture Duration

To compare the fold changes of the heart signature genes between hPSC-CMs on day t and adult CMs on day $t^{*}$, the Pearson correlation coefficient $r_{{ϒ}_{t},{ϒ}^{*}}$ has been calculated during the hPSC-CMs differentiation for two random variables ${ϒ}_{t}$ and ${ϒ}^{*}$.

(2) $$r_{{ϒ}_{t},{ϒ}^{*}}=\frac{cov_{{ϒ}_{t},{ϒ}^{*}}}{\delta_{{ϒ}_{t}}\delta_{{ϒ}^{*}}}=\frac{\sum_{i\in N}\left(y_{i}^{t}-\bar{{ϒ}_{t}}\right)\left(y_{i}^{*}-\bar{{ϒ}^{*}}\right)}{\sqrt{\sum_{i\in N}{\left(y_{i}^{t}-\bar{{ϒ}_{t}}\right)}^{2}\sum_{i\in N}{\left(y_{i}^{*}-\bar{{ϒ}^{*}}\right)}^{2}}},$$

where cov is the covariance and σ is the standard deviation. N=203 is the sample size, which equals the total number of sample cardiac genes indexed by i. For hPSC-CMs on day t during in vitro differentiation, $\bar{{ϒ}_{t}}=\left(\sum_{i\in N}y_{i}^{t}\right)\text{/}N$ is the sample mean of the 203 genes. For adult CMs, $\bar{{ϒ}^{*}}=\left(\sum_{i\in N}y_{i}^{t}\right)\text{/}N$ is the corresponding sample mean.

Pearson correlation coefficient is a measure of the similarity of two random variables between −1 and 1. If ${ϒ}_{t}$ and ${ϒ}^{*}$ are highly correlated, i.e., $\left|r_{{ϒ}_{t},{ϒ}^{*}}\right|$ is close to 1, it is reasonable to build a model such that ${ϒ}_{t}$ can be predicted based on the value of ${ϒ}^{*}$. An example relationship between the response ${ϒ}_{t}$ and the predictor ${ϒ}^{*}$ can be described as a linear function:

(3) $${\hat{ϒ}}_{t}=\alpha_{t}+\beta_{t}{ϒ}^{*},\;\forall t\in T,$$

where ${\hat{ϒ}}_{t}$ is the predicted response of ${ϒ}_{t}$. The coefficients $\alpha_{t}$ and $\beta_{t}$ are the intercept and slope, respectively. $\beta_{t}$ tells how much the dependent variable ${\hat{ϒ}}_{t}$ is expected to increase when the independent variable ${ϒ}^{*}$ increases by one.

For the simple linear regression model in Equation (3), $\alpha_{t}$ and $\beta_{t}$ are selected to minimize the difference between the predicted response ${\hat{ϒ}}_{t}$ and the measured response ${ϒ}_{t}$. In particular, to quantify how much variance remains after fitting the linear model, the sum of squared error $\left(\text{SSE}\right)$ is adopted to measure the performance of the predictive model.

(4) $$SSE=\sum_{i\in N}{\left(y_{i}^{t}-{\hat{ϒ}}_{t}\right)}^{2},$$

where SSE is measured by the squared differences between the predicted and actual target values. To minimize SSE, the coefficients have the following values.

(5) $$\left\{\begin{array}{l} \beta_{t}=r_{{ϒ}_{t},{ϒ}^{*}}\frac{\delta_{{ϒ}^{*}}}{\delta_{{ϒ}_{t}}},\\ \alpha_{t}=\bar{{ϒ}_{t}}-\beta \bar{{ϒ}^{*}}, \end{array}\right.$$

where the above coefficients are derived by setting the partial derivative of SSE to 0. As compared with slope $\beta_{t}$, $r_{{ϒ}_{t},{ϒ}^{*}}$ can be treated as the standardized slope of the simple linear regression model.

Meanwhile, the coefficient of determination, $R_{{ϒ}_{t},{ϒ}^{*}}^{2}$, measures that “$R_{{ϒ}_{t},{ϒ}^{*}}^{2}\times 100\%$ of the variation in ${ϒ}_{t}$ is reduced by taking into account predictor ${ϒ}^{*}$”. $R_{{ϒ}_{t},{ϒ}^{*}}^{2}$ tells how much of the total variance can be explained by the linear model.

(6) $$R_{{ϒ}_{t},{ϒ}^{*}}^{2}=1-\frac{SSE}{SST}=1-\frac{SSE}{\sum_{i\in N}{\left(y_{i}^{t}-\bar{{ϒ}_{t}}\right)}^{2}},$$

where the sum of squared total $\left(\text{SST}\right)$ quantifies the total variance of the target outcome as the sum of squared distance between individual data point $y_{i}^{t}$ and the mean of the response variable $\bar{{ϒ}_{t}}$. Note that for the linear regression model in Equation (3), $R_{{ϒ}_{t},{ϒ}^{*}}^{2}={\left(r_{{ϒ}_{t},{ϒ}^{*}}\right)}^{2}$.

**Figure 2.**
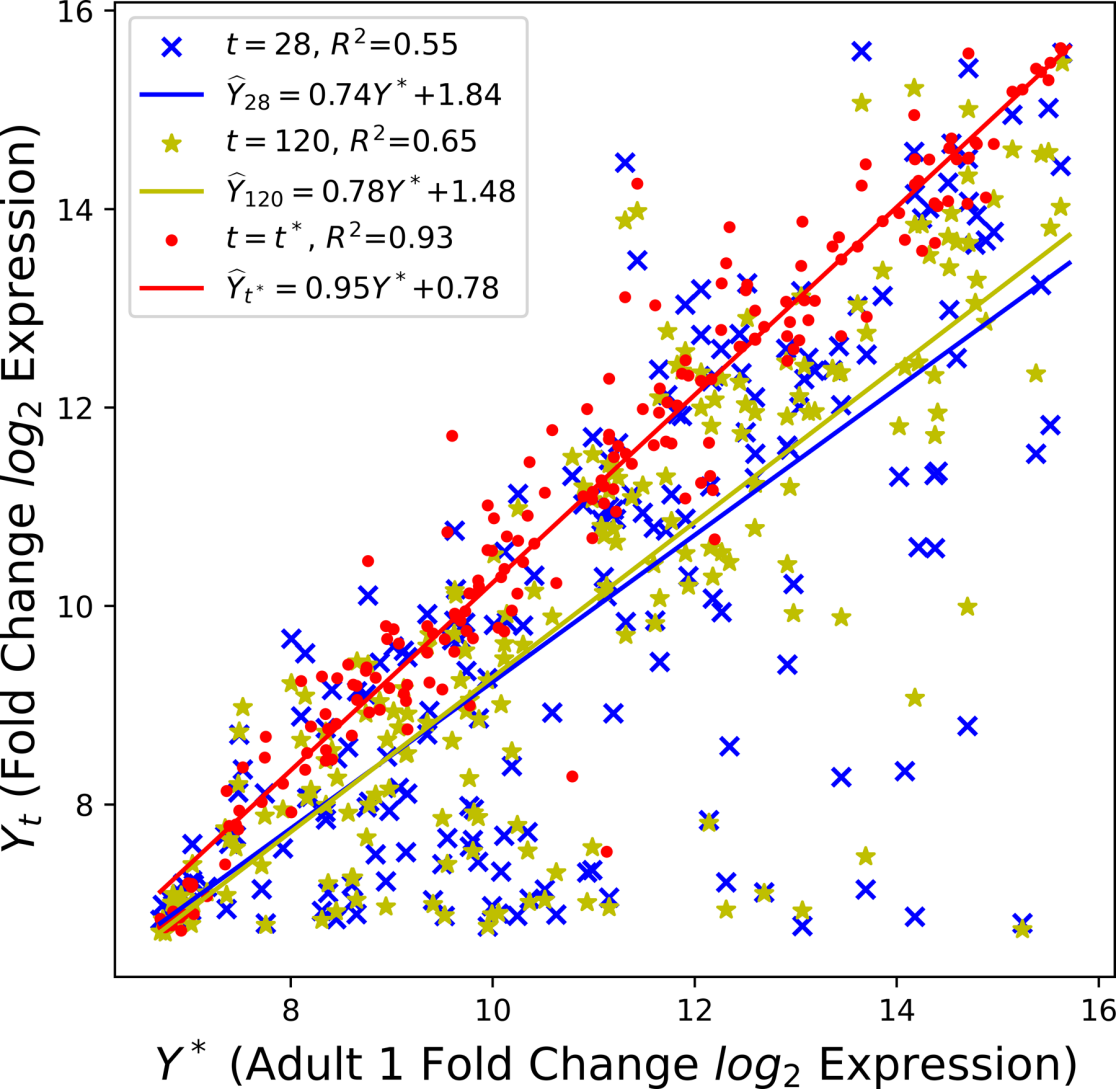
Comparison of the cardiac-specific genes between adult CMs and hPSC-CMs.

As illustrated in Table 2, $R_{{ϒ}_{t},{ϒ}^{*}}^{2}$ increases with culture time t. In particular, the squared Pearson correlation coefficient $R_{{ϒ}_{28},{ϒ}^{*}}^{2}=0.55$ shows a moderate positive linear relationship between the gene expression profiles of day 28 hPSC-CMs and adult CMs. Meanwhile, the value of 93.0% in Figure 2 suggests a strong linear relationship between two adult samples, where only 7% of the variation in the adult 2 sample is left to explain after taking into account the adult 1 sample in a linear way. However, knowing the hPSC-CMs are continually becoming mature is not sufficient. The quantification of the mature stage will be discu d next.

## 4. Data-Driven Maturity Quantification

**Figure 3.**
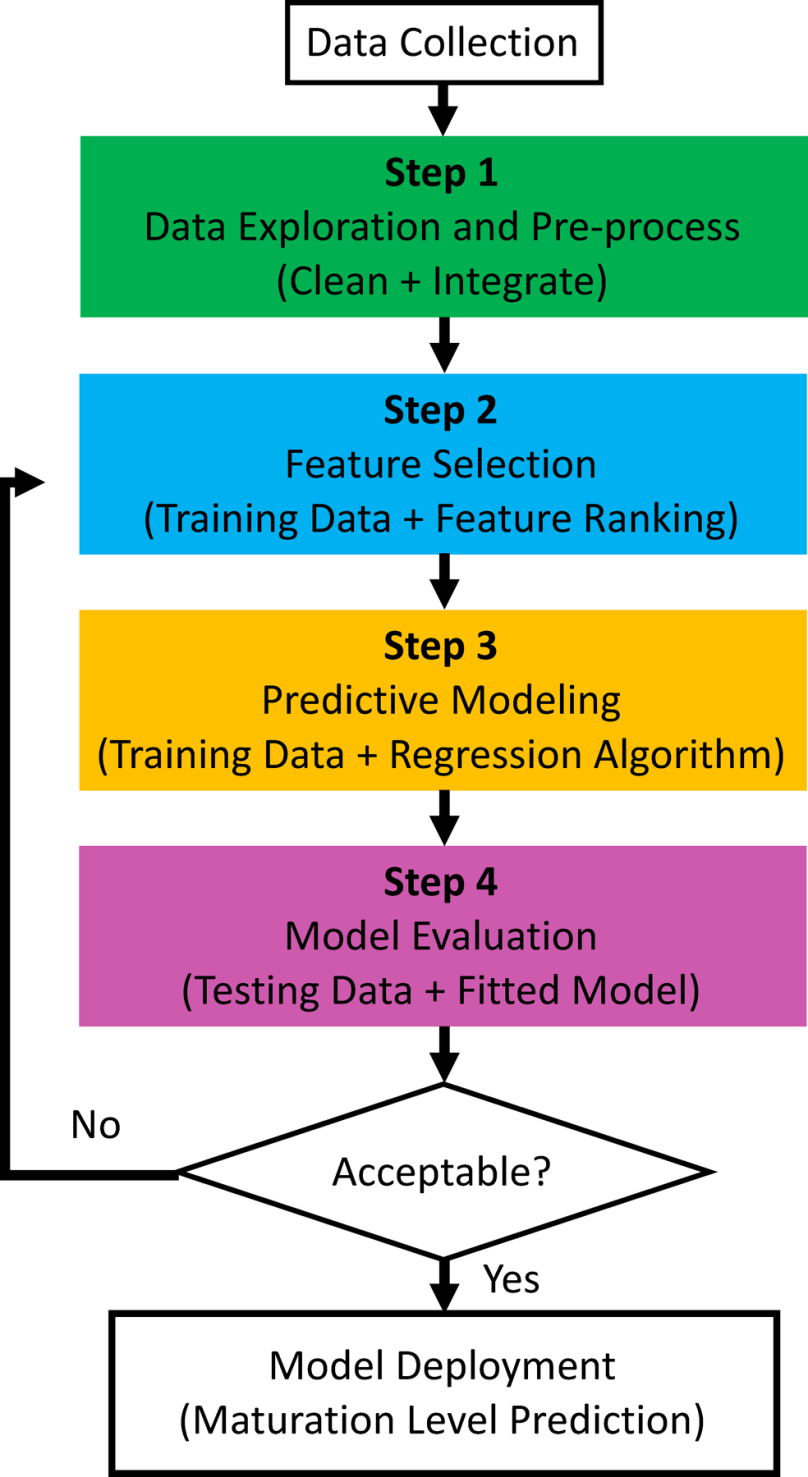
The data-driven maturation quantification pipeline.

Aiming for an effective algorithm to quantify the maturity level of hPSC-CMs, the data-driven pipeline in Figure 3 is designed to select cardiac-specific genes and then map those genes to the in vitro culture time t, which is adopted as the proxy of the maturity stage.

### 4.1. Data-Driven Maturity Quantification Pipeline

Data Collection: We collected two publicly available datasets with gene-specific fold change values across 12 in vitro culture time points in T. The first dataset has recorded 203 cardiac genes for one group of cells (Supp_table4 in [25]), and the second dataset has 48,804 genes recorded for three groups of cells (GSM873339-GSM873374 in [16]).

Step 1: Since both datasets are collected in the same culturing environment setting, to integrate them and increase the data volume, the gene “ID” (used by dataset 2) is mapped to the “ILMN_Gene” (used by dataset 1) according to the GPL6884 table in [27], which can translate the “unique identifier for the probe” to the “internal gene symbol”. Since multiple IDs can map to the same ILMN_Gene, we remove the redundant entries in dataset 2.

After cleaning dataset 2 and taking the intersection of gene symbols in both datasets, the resulting integrated dataset has 48 data records: four groups of cells across 12 in vitro culture time points. For each record, 189 genes listed in Table A1 in Appendix A are recorded. For notation simplicity, the genes that are fed into the next steps (feature selection and predictive modeling) are denoted as $\text{X}=\left\{x_{i}\;|\;i\in N_{c}\right\}$, where $x_{i}$ represents the fold change value of the i-th cardiac gene in $N_{c}=\left\{1,\;\ldots ,\;189\right\}$.

Steps 2–3: With 48 records of $\left\{\text{X},\;T\right\}$, the train–test split in Table 3 separates the total dataset into two parts: $M_{train}=\left\{1,\;\ldots ,\;M_{train}\right\}$ records for training and the remaining $M_{test}=\left\{1,\;\ldots ,\;M_{test}\right\}$ for testing. Based on the training data, a subset of X is selected such that regression analysis can be conducted to learn a function f and predict T.

(7) $$\hat{T}=f\left({\text{X}}^{\prime }\right),\;{\text{X}}^{\prime }\subseteq \text{X},$$

where $\hat{T}$ is the time estimated by the predictive model f.

The purpose of feature selection is to remove unnecessary, irrelevant, and redundant genes. The remaining important cardiac genes in ${\text{X}}^{\prime }$ are useful to create an accurate predictive model f, which aims to minimize the root-mean-square error (RMSE).

(8) $$f=\arg\;\min\;RMSE\left(T\right)=\arg\;\min\sqrt{\frac{1}{M_{train}}SSE\left(T\right)},$$

where for the j-th training record, $T_{j}$ is the ground truth, and ${\hat{ϒ}}_{t}$ is the corresponding estimated value. $SSE\left(T\right)=\sum_{j\in M_{train}}{\left(T_{j}-{\hat{T}}_{j}\right)}^{2}$ is the sum of squared errors of predicting T, which measures the deviation between the observed value and the true value.

Note that after splitting the training data and test data, the standardization of numerical features is performed over training data, such that each gene’s fold change value has zero mean and unit variance.

(9) $$x_{i,j}^{Normalized}=\frac{x_{i,j}-{\bar{x}}_{i}}{\sigma_{x_{i}}},\;\forall j\in M_{train}\cup M_{test},$$

where $x_{i,j}$ is the fold change value of the i-th gene in the j-th collected record. For each cardiac gene, based on all necessary statistics of the training data (mean ${\bar{x}}_{i}=\sum_{j\in M_{train}}x_{i,j}\text{/}M_{train}$ and standard deviation $\sigma_{x_{i}}$), the standardization is also performed on test data. For notation simplicity, we dropped the superscript *Normalized* in the rest of the paper.

With all of the numerical values in the dataset being standardized, no gene can dominate the objective function and make the estimator unable to learn from other features correctly as expected.

Step 4: To verify the performance of the feature selection algorithm and predictive modeling, the standardized data records in the test dataset are fed into the model, and the corresponding RMSE and $R^{2}$ score are adopted as the performance evaluation metrics.

(10) $$R_{T,\hat{T}}^{2}=1-\frac{\sum_{j\in M_{test}}{\left(T_{j}-{\hat{T}}_{j}\right)}^{2}}{\sum_{j\in M_{test}}{\left(T_{j}-{\bar{T}}_{test}\right)}^{2}},$$

where ${\bar{T}}_{test}=\left(\sum_{j\in M_{test}}T_{j}\right)\text{/}M_{test}$ is the average ground truth culture time of the testing data. $R^{2}$ is the fraction of the total sum of squares that is explained by the regression, and the closer $R^{2}$ is to 1, the better the model.

Since Steps 1 and 4 are fixed for the proposed pipeline, to improve the performance of the hPSC-CM culture time prediction, the following feature selection algorithms (Step 2) and predictive modeling (Step 3) are investigated in our study.

- Method 1 (**M**_1_): Filter method and linear regression;
- Method 2 (**M**_2_): Wrapper method;
- Method 3 (**M**_3_): Embedded method;
- Method 4 (**M**_4_): Non-linear feature selection and non-linear regression;
- Method 5 (**M**_5_): Non-linear feature selection and linear regression.

### 4.2. M_1_—Filter Method (Pearson Correlation) + Linear Regression

To select the dominant cardiac genes in $N_{c}$ that determine the culture duration T, the univariate feature selection technique examines the linear strength of the relationship (such as Pearson correlation coefficient) between each input feature $x_{i}$ and the corresponding output variable T. The ranking of input features based on their strength of relationship concerning the output variable can gain a preliminary understanding of the collected data.

The detailed steps of the correlation-based filter method include the following. (1) The constant and quasi-constant cardiac genes in X, which have variance less than the threshold 0.01, i.e., ${\left(\delta_{x_{i}}\right)}^{2}<0.01$, are removed from $N_{c}$. This step can delete the cardiac genes that do not change significantly when the culture time T increases. (2) Irrelevant cardiac genes with the absolute Pearson correlation coefficient less than the threshold 0.5, i.e., $|r_{x_{i},T}|<0.5$ are removed as well, as they contain little information to predict the output T. (3) When the correlation coefficient for a pair of cardiac genes $\left(x_{i_{1}},x_{i_{2}}\right)$ is higher than the threshold 0.5, i.e., $|r_{x_{i_{1}},x_{i_{2}}}|>0.5$, only one gene with a higher correlation with the output variable T is kept.

**Figure 4.**
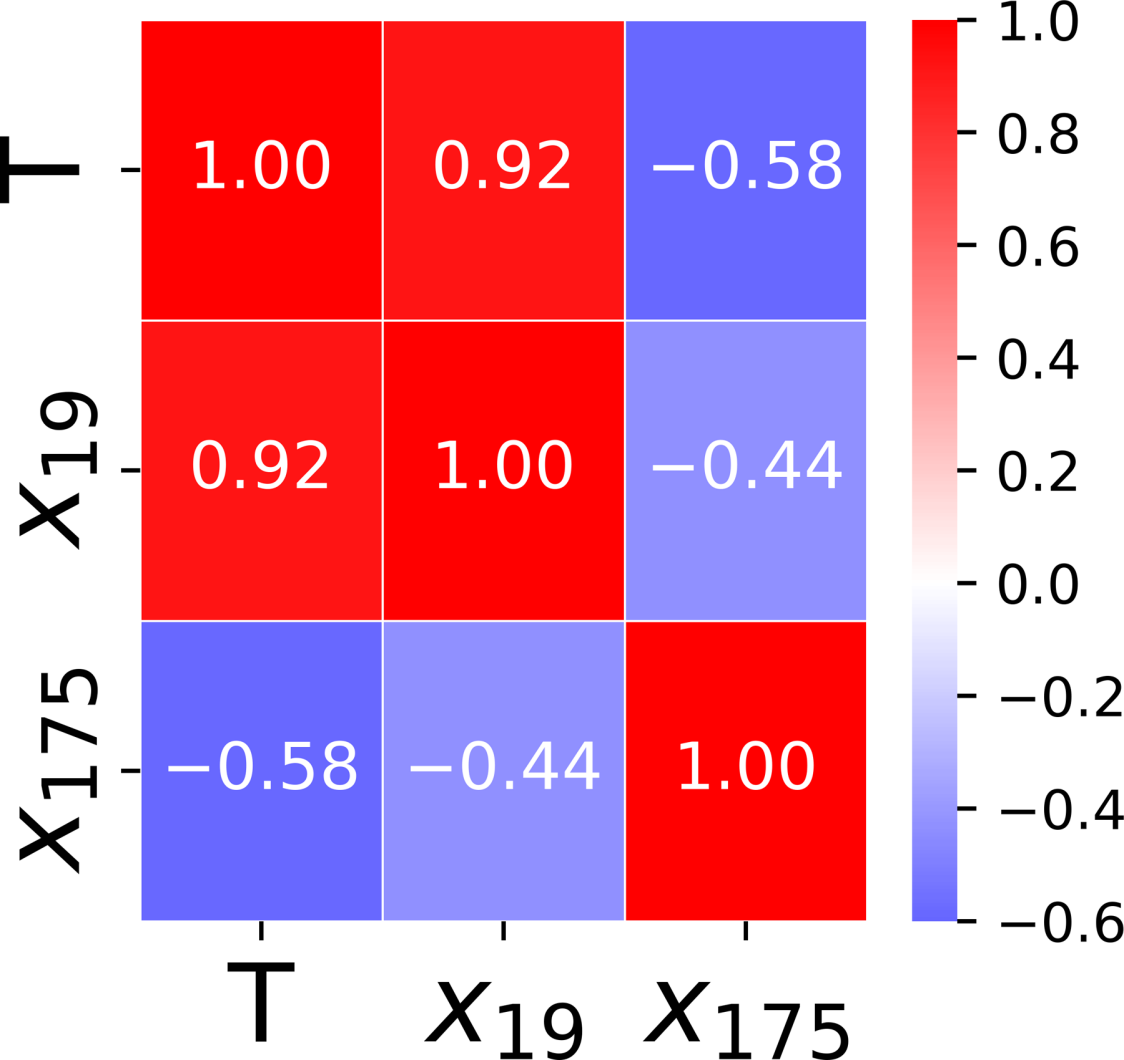
Pair-wise correlation of the gene selected by **M**_1_.

As illustrated by the heatmap in Figure 4, $k^{*}=2$ cardiac genes are kept by the filter method, and each selected input feature $\left({\text{X}}^{\prime }=\left\{x_{19},x_{175}\right\}\right)$ has a high correlation to the output T. The correlations among the selected features are less than the predefined threshold.

After the above feature selection steps, the multiple linear regression is applied to the clean dataset and the coefficient of each feature in the linear function $f_{1}$ is obtained as follows:

(11) $$\hat{T}=f_{1}\left(x_{19},x_{175}\right)=\sum_{i\in \left\{19,175\right\}}\beta_{i}x_{i}+\alpha =30.42x_{19}-8.32x_{175}+37.67,$$

where the coefficients of the above linear function are selected to minimize RMSE in Equation (8). The intercept is 37.67 and other coefficients show the change in the output variable $\hat{T}$ for one unit of change in the input cardiac gene while holding other input genes in the model constant. Since $\left|\beta_{19}|\;>\;|\beta_{175}\right|$, $x_{19}$ has more impact on the culture time prediction as compared to $x_{175}$.

### 4.3. M_2_—Wrapper Method (Recursive Feature Elimination + Linear Regression)

Different from **M**_1_ with sequential feature selection and regression modeling, the wrapper method uses the performance of the regression model as evaluation criteria for the feature selection scheme. In particular, the wrapper method can generate different subsets of features, and each subset is used to build a model and train the learning algorithm. The subset yielding the best performance in terms of RMSE is selected as the final features.

During the subset generation process, the recursive feature elimination (RFE) method involves multiple rounds of elimination of the input variables. In each round, RFE specifies the number of input features that should be selected to build the regression model, and then performs recursive feature elimination. The detailed procedure is given below.

Iteration k: The number of features being selected is iterated from k=1 to $k=min\left\{\left|N_{c}|,\;|M_{train}\right|\right\}=40$, where |•| means the cardinality of the cardiac genes in set $N_{c}$ and the cardinality of the training set $M_{train}$. Note that the number of features being selected cannot exceed the number of records in the training dataset.

In the k-th iteration, RFE performs the following two steps.

Step **M**_**2**.**1**_: Build a full linear regression model with all of the input features in the cardiac gene set $N_{c}$.

(12) $$\hat{T}=f_{2}\left(\text{X}\right)=\sum_{i\in N_{c}}\beta_{i}x_{i}+\alpha ,$$

where the above model aims to minimize the RMSE in Equation (8). Note that since we are trying to build a model with $|N_{c}|=\text{189}$ coefficients based on $|M_{train}|=40$ records in the training dataset, the full model is not unique because of the small dataset: $\left|N_{c}|>|M_{train}\right|$.

Step **M**_**2**.**2**_: Rank features based on the absolute value of the coefficient. The least important feature is pruned from the current set of cardiac features. Note that since the training dataset has been standardized to unit variance, the importance of a feature increases with increasing $\left|\beta_{i}\right|$. Otherwise, the feature importance ranking has to take account of the standard deviation of each cardiac gene as well: $|\beta_{i}|\sigma_{x_{i}}$[28].

Ending Condition for the k-th iteration: For each k-th iteration, repeat the feature elimination Steps **M**_**2**.**1**_–**M**_**2**.**2**_ over the pruned set until the number of genes left is k.

**Figure 5.**
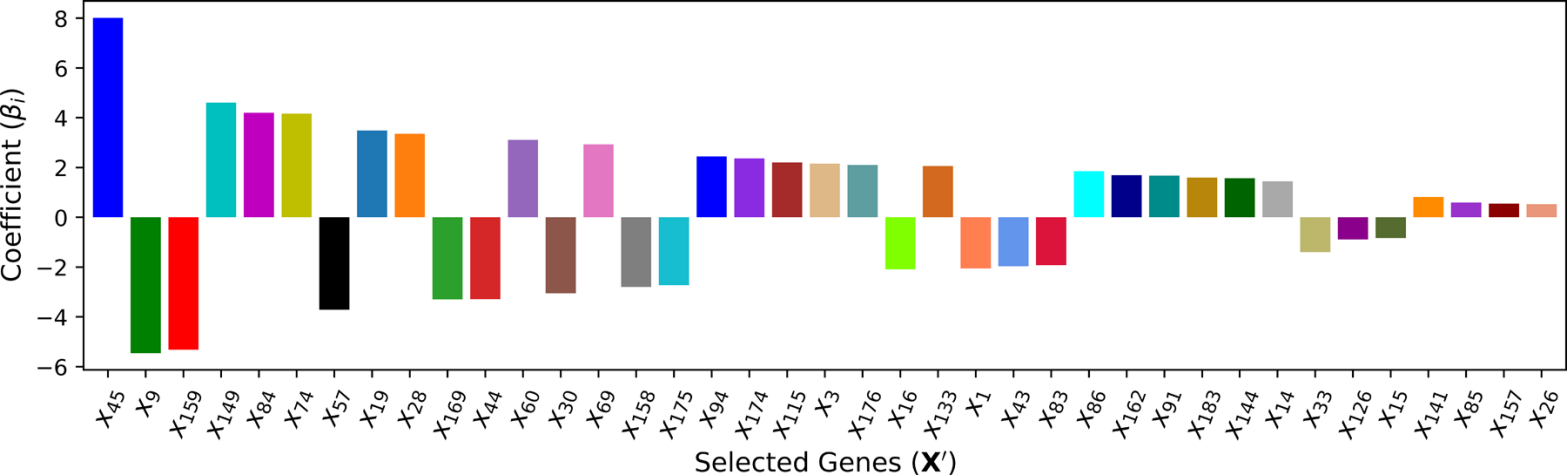
The ranking of cardiac genes selected by **M**_2_.

After all of the iterations are completed $\left(k=40\right)$, the subset size $k^{*}$ that optimizes the performance criteria (smallest RMSE) is used to select the input variables, and the corresponding optimal subset is then used to train the final model. As illustrated in Figure 5, $k^{*}=39$ selected features are ranked based on the absolute linear regression coefficient $\left|\beta_{i}\right|$ in the final model.

### 4.4. M_3_—Embedded Method (Lasso Regularization)

Embedded methods combine the qualities of the filter and wrapper methods. It is implemented by regression algorithms with built-in feature selection schemes. Regularization is the most commonly used embedded method that introduces additional constraints to bias the regression model toward less input cardiac features. As shown below, the least absolute shrinkage and selection operator (LASSO), i.e., **L1** regularization for generalized linear models, has an additional weighted penalty against the complexity of the model.

(13) $$f_{3}=\arg\;\min\left\{\sum_{j\in M_{train}}{\left(T_{j}-{\hat{T}}_{j}\right)}^{2}+\lambda \sum_{i\in N_{c}}\left|\beta_{i}\right|\right\},$$

where $\hat{T}=f_{3}\left(\text{X}\right)=\sum_{i\in N_{c}}\beta_{i}x_{i}+\alpha$. The model complexity is the sum of the absolute coefficients for all the input features. Meanwhile, 0≤λ≤∞ is adjustable and the higher the value, the more the coefficient $\beta_{i}$ is forced to shrink. With this penalty term, if a feature $x_{i}$ is irrelevant, LASSO penalizes its coefficient and makes it 0, and this feature is then removed from the dataset.

**Figure 6.**
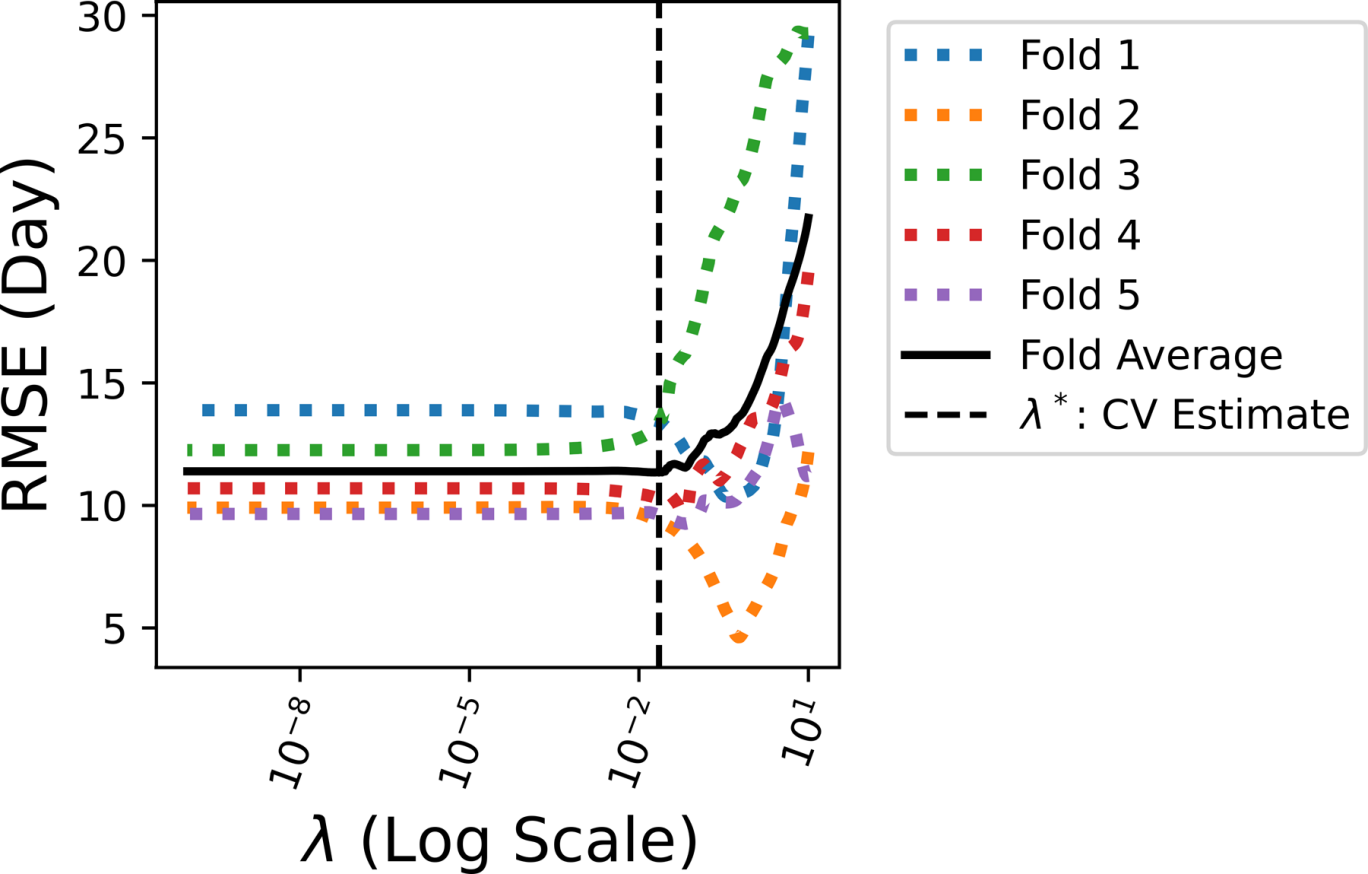
The tuning of regularization coefficient λ.

**Figure 7.**
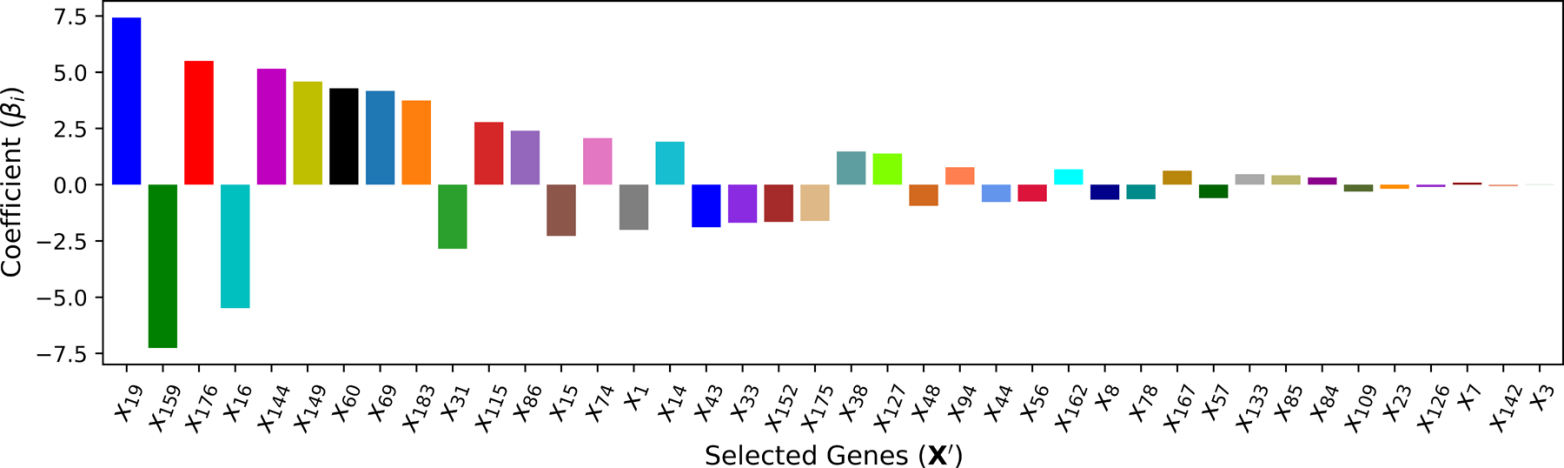
The ranking of cardiac genes selected by **M**_3_.

To determine the appropriate value for λ, cross-validation (CV) is adopted and the training dataset is divided into 5 non-overlapping folds. A total of 100 values of λ are iterated to train Lasso models over 5 folds. As illustrated in Figure 6, the optimal $\lambda^{*}$ yields the best average performance in terms of the smallest fold average RMSE: $\lambda^{*}=0.0226$. With the chosen optimal $\lambda^{*}$, $k^{*}=40$ features with non-zero coefficient $\beta_{i}$ in the corresponding predictive model are ranked in Figure 7.

### 4.5. M_4_—Non-Linear Feature Selection and Regression (XGBoost Method)

When the regression model f in Equation (7) represents a non-linear relationship between the input cardiac genes and the output culture time, the tree-based methods, e.g., decision trees, random forest, and extreme gradient boosting (XGBoost) [29], can be applied to perform feature selection with low complexity. They can model non-linear relations well and do not require much tuning.

**Figure 8.**
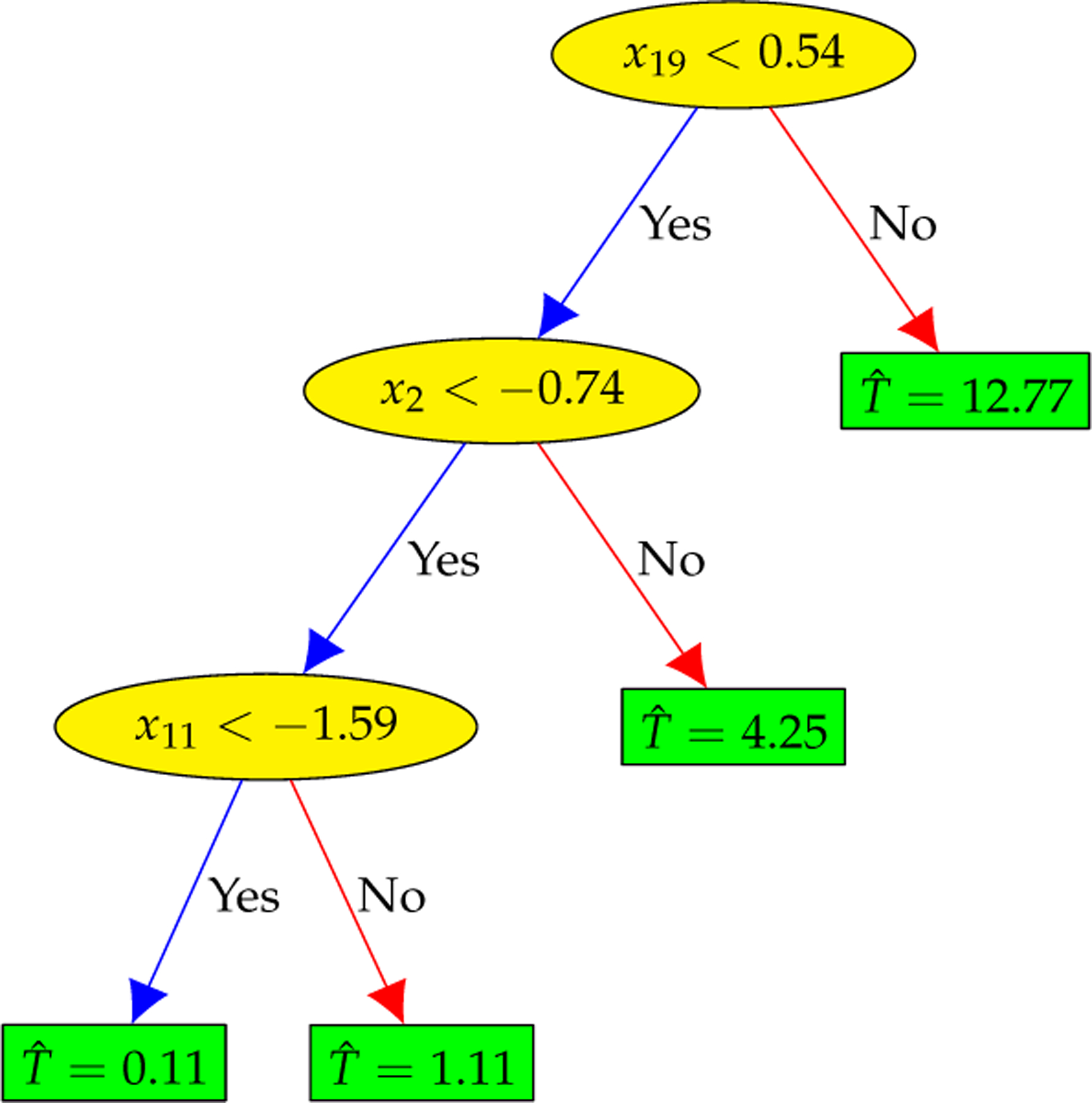
The first decision tree $f_{4}^{1}$ of XGBoost.

As illustrated in Figure 8, the decision tree can create a regression model that predicts the culture time T by evaluating a sequence of true/false questions regarding the cardiac features. Although an effective regression model, the decision tree is very sensitive and small changes to the training set can result in significantly different tree structures.

XGBoost addre s this issue by leveraging the wisdom of crowds wherein a large number of individual trees operating as a committee will outperform any of the individual constituent trees.

(14) $$\hat{T}=f_{4}\left(\text{X}\right)=\sum_{b=1}^{B}f_{4}^{b}\left(\text{X}\right),\;f_{4}^{b}\in F,$$

where B is the number of trees. $f_{4}^{b}$ is a function in the functional space F, and F is the set of all possible trees, which can map the data record to the green leaf node. B is a tunable parameter that can be iterated to reduce the training RMSE.

**Figure 9.**
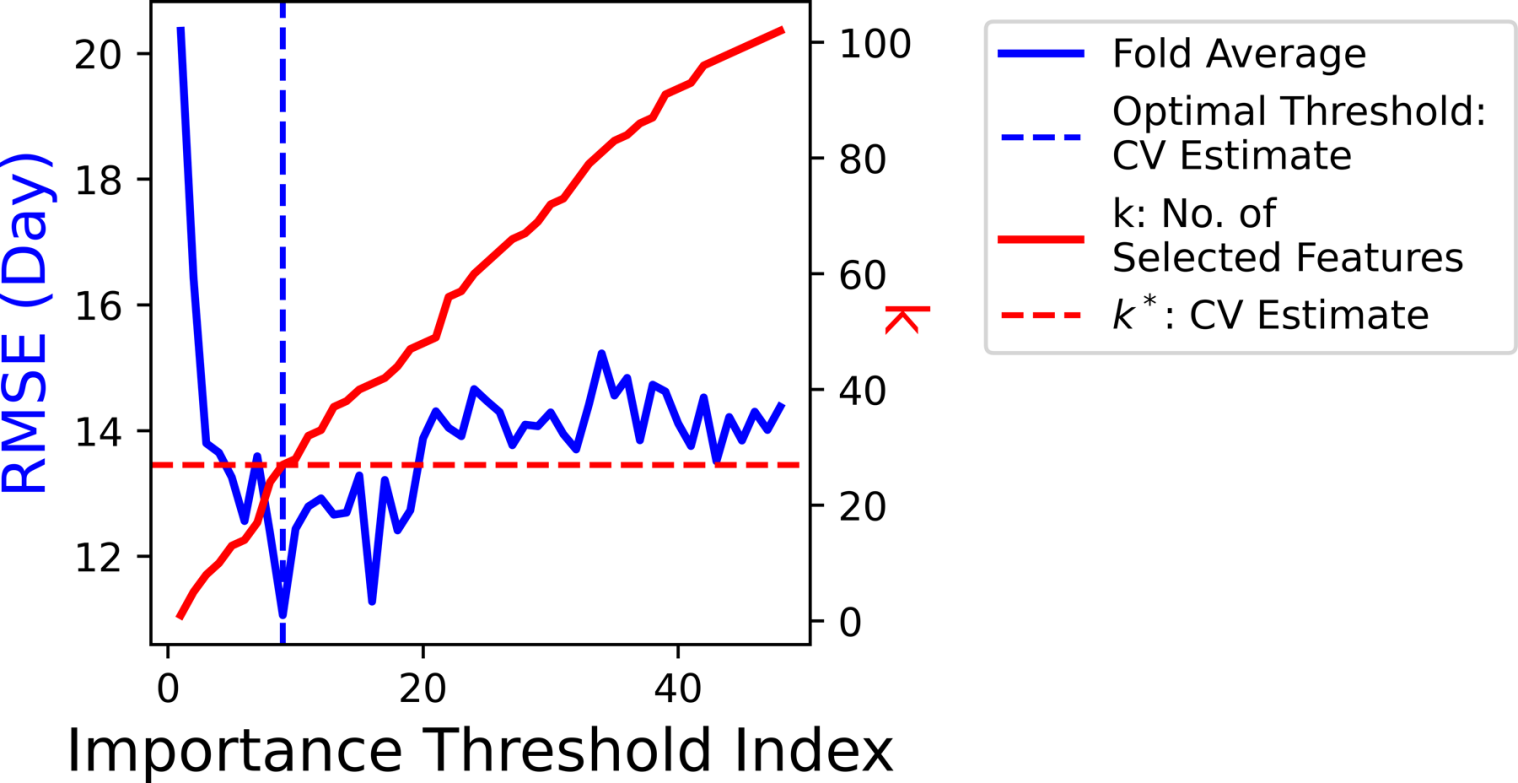
XGBoost-based feature selection.

**Figure 10.**
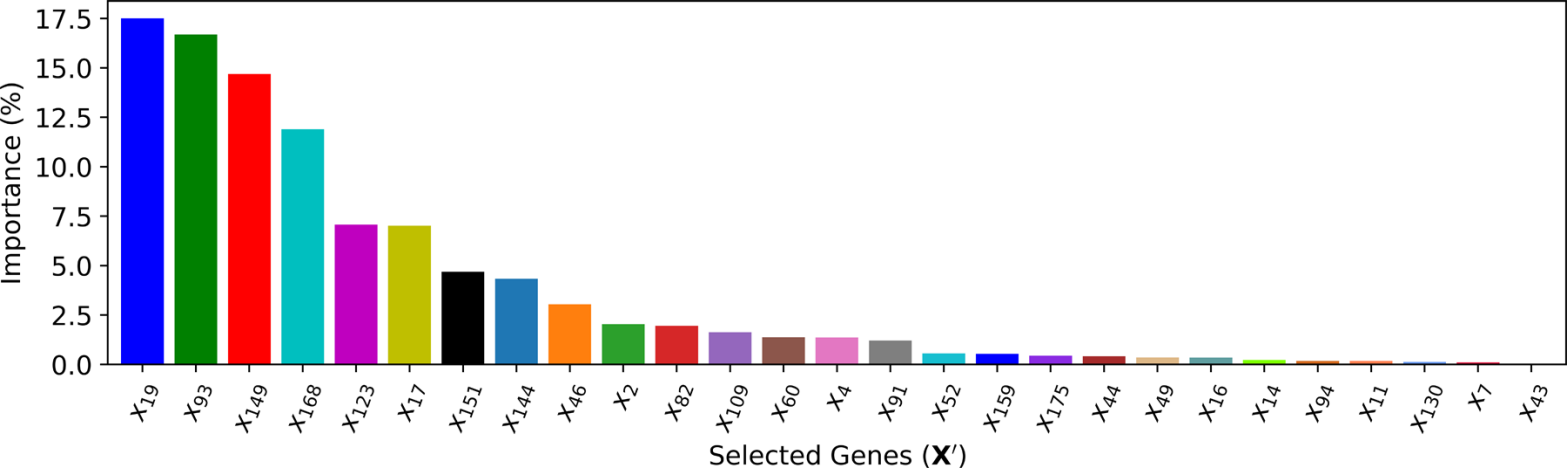
The ranking of cardiac genes selected by **M**_4_.

The importance of a feature in the XGBoost can be measured as the number of times a feature is used to split the data across all trees. With this feature ranking mechanism, multiple XGBoost models are trained iteratively by feeding the top k features. As shown in Figure 9, the cross-validation performance shows that the best XGBoost model only needs the top $k^{*}=27$ features. When feeding the selected top 27 cardiac genes, various parameters in the XGBoost model are tuned based on the training data, and the resulting feature importance is ranked in Figure 10.

### 4.6. M_5_—XGBoost Method + Linear Regression

**Figure 11.**
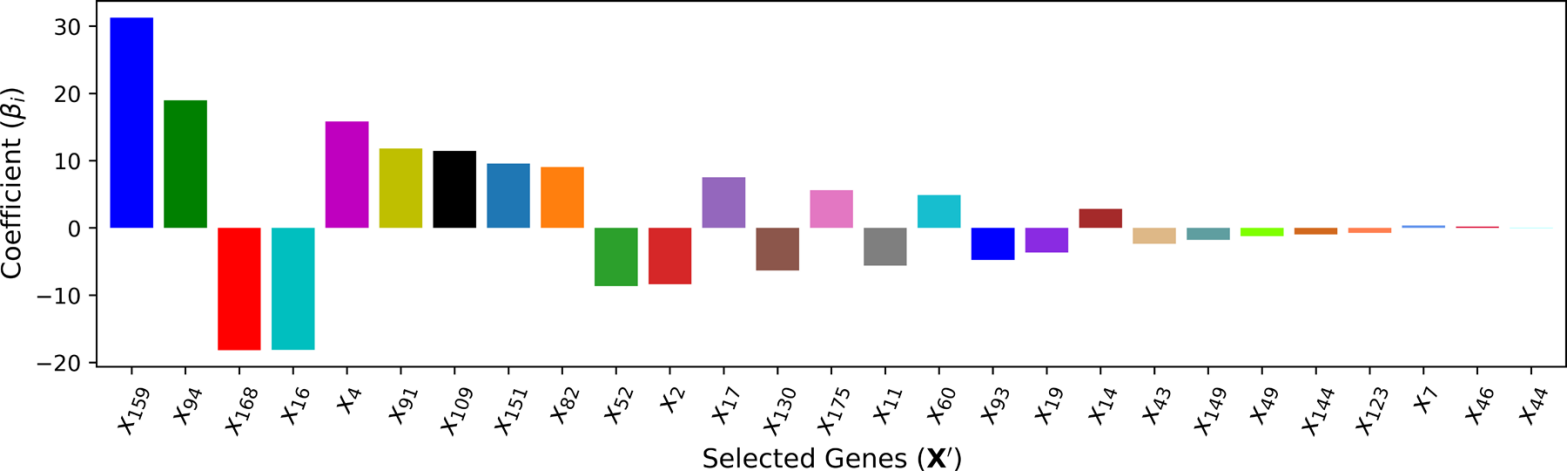
The ranking of cardiac genes used by **M**_5_.

**M**_5_ adopts the input features selected by **M**_4_ (XGBoost) and predicts the output with the linear regression model. This method is introduced to measure the improvement that **M**_4_ achieves by capturing nonlinearity among input features and output T. In other words, this method signifies the impact and importance of implied nonlinearity among the input features and between pairs of inputs and output. The coefficients of the selected cardiac genes in the linear regression model are shown in Figure 11.

## 5. Results

The five feature selection and regression model development methods (**M**_1_ to **M**_5_) are applied to select the most important cardiac-specific genes that are related to the hPSC-CM culture duration. With the selected genes, predictive algorithms are adopted to quantify the maturity level of hPSC-CMs. The resulting performance over the test data is collected.

### 5.1. Culture Time Prediction

**Figure 12.**
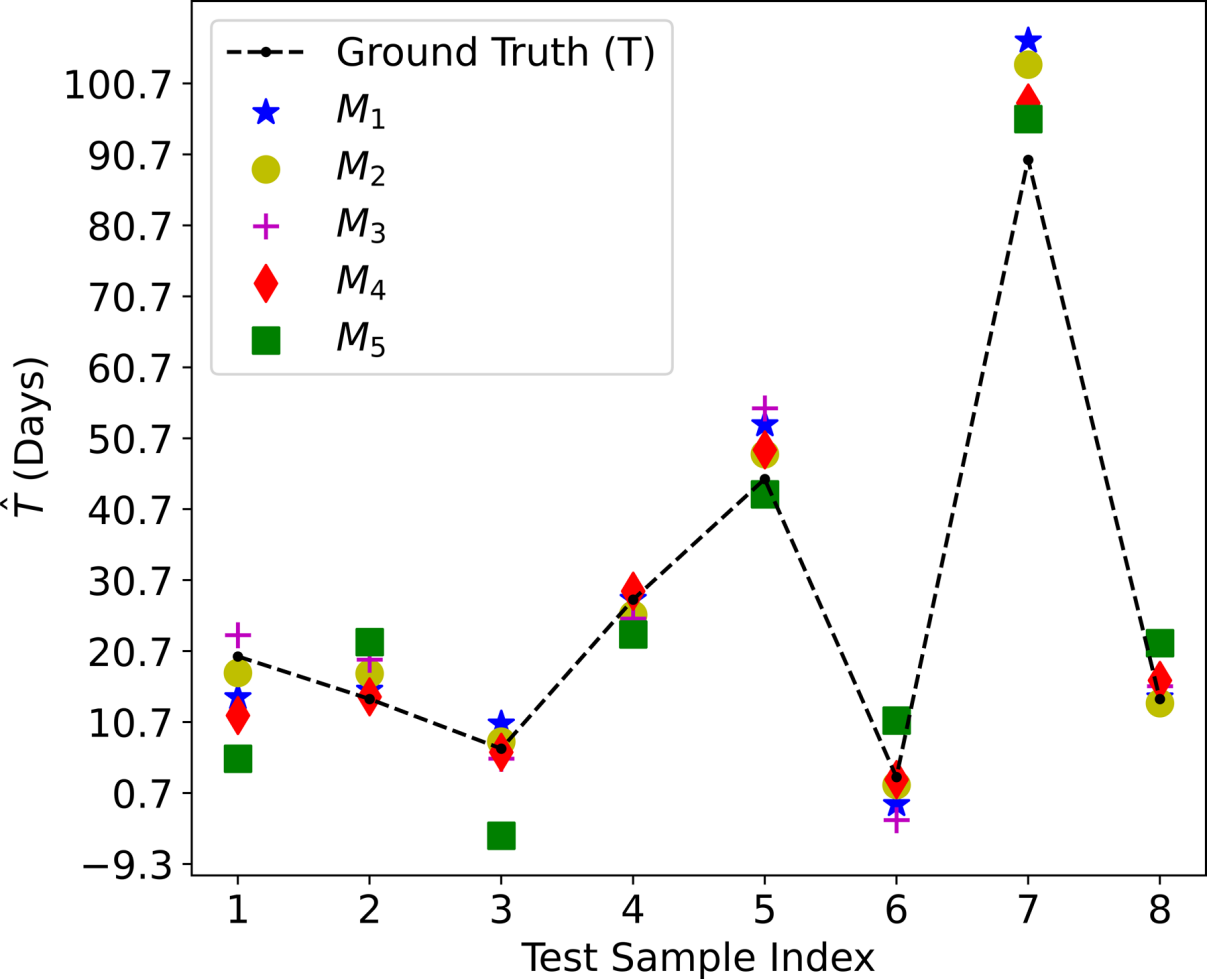
The predicted maturity level.

The comparison between the observed culture time T and the predicted culture time $\hat{T}$ over the test dataset is shown in Figure 12. All of the five methods have acceptable test performance in terms of a small discrepancy between T and $\hat{T}$, as verified by the low RMSE and the $R^{2}$ score closer to 1 in Table 4.

For the linear regression models, **M_2_** (RFE with 39 features) yields the best performance, which is followed by **M_3_** (Lasso with 40 features), **M_1_** (Pearson correlation with 2 features), and **M_5_** (with 27 features selected by XGBoost). For these four linear models, the predictive performance does not strictly grow or decrease with the number of selected cardiac genes. The reason is that on one hand, the predictive performance will improve when the number of important cardiac genes being selected increases. On the other hand, the performance will drop when less important cardiac genes are included in the predictive model.

Moreover, the non-linear model **M_4_** (XGBoost) outperforms all of the four linear models. This is another successful application of XGBoost because it can extract useful predictive information from 27 cardiac genes while **M_2_** (RFE) needs 39 cardiac genes. The performance degradation from **M_4_** to **M_5_** verifies that XGBoost can capture the non-linearity correlation among the selected cardiac genes and culture time.

### 5.2. Cardiac Gene Selection

As illustrated in Table 4, three methods (**M_1_**, **M_3_**, and **M_4_**) have selected $x_{19}$ as the top gene in ${\text{X}}^{\prime }$ that correlates with the culture duration. The detailed importance level of $x_{19}$ can be found in Equation (11), Figures 7 and 10. Although **M_2_** (RFE in Figure 5) ranks $x_{19}$ as the eighth most important gene, the training dataset is very small (only 40 data records), and there is randomness in the 189 coefficients of the full model returned by Equation (12). Consequently, the ranking of the cardiac genes returned by **M_2_** has randomness as well. When $x_{19}$ is not ranked as the top cardiac gene, **M_5_** has the worst performance. It is thus reasonable to conclude that $x_{19}$ is the most important gene in terms of predicting the culture time of hPSC-CMs.

In addition, biology domain knowledge suggests that $x_{19}$ (Gene *CASQ2*: Calsequestrin 2) triggers skeletal and cardiac muscle contraction and plays a critical role in excitation–contraction coupling in the heart and in regulating the rate of heartbeats. This validates our proposed data-driven approach for assessing hPSC-CM maturity in in vitro culture systems. Moreover, this observation is particularly significant as it can inform the design of maturation promotion schemes for hPSC-CMs. By stimulating hPSC-CMs in a way that accelerates the fold-change increase in *CASQ2*, it is likely to facilitate their maturation into a more adult-like phenotype.

## 6. Discussion

We have proposed an innovative approach that integrates machine learning with biological data to evaluate hPSC-CM maturity. By correlating publicly available gene expression profiles with culture duration, our data-driven framework represents a significant improvement over traditional biological qualitative techniques in Figure 1.

### 6.1. Limitations and Future Work for Maturity Evaluation

Although the data-driven pipeline is novel, the limited data size used in this study increases sensitivity to noise and outliers in the gene profile. This issue is further complicated by the inherent variability in cell maturation rates, as cells may achieve similar functionalities at different paces. As a result, the correlation between post-differentiation culture time and unobservable maturity level may not be perfect. These factors could cause the regression model to overfit the current dataset, thereby limiting its generalizability to other datasets.

To address these limitations, future work will aim to enhance the robustness and generalizability of the proposed approach by improving both data quantity and model design through the following strategies: (1) Collecting additional experimental data to further validate the regression model’s predictions. For example, predicted maturity levels could be compared with functional assays or the physiological characteristics of cardiomyocytes to improve the model’s accuracy and reliability. (2) Exploring more reliable proxies for maturation levels and developing advanced machine learning models capable of uncovering latent maturity levels from relatively small datasets. These efforts will further strengthen the predictive framework and enhance the model’s applicability and performance.

### 6.2. Limitations and Future Work for Maturity Monitoring and Promotion

Since the cultured cells are destroyed during data acquisition, the existing gene expression-based hPSC-CM maturity quantification schemes are invasive and costly, similar to the morphology- and structure-based schemes and calcium handling-based schemes described in Section 2. For non-invasive maturity quantification, the contractile function and electrophysiological property-based approaches have the most potential, because advanced lab-on-chip devices [30] can be designed and fabricated to monitor the properties that correlate with cell maturity indicator, such as gene $x_{19}$, in real time as the culture duration is prolonged.

Meanwhile, by leveraging a lab-on-chip device capable of real-time cell status monitoring and potential stimulation through mechanical stretching or electrical pulsing, future longitudinal studies can utilize live data to track the maturation process over time. This approach could offer deeper insights into the maturation dynamics of hPSC-derived cardiomyocytes, including adaptive stimulation adjustments.

## 7. Conclusions

We have proposed a data-driven pipeline for evaluating the maturity of hPSC-CMs. Using public gene expression data spanning various in vitro culture time points, we demonstrated the feasibility of using culture time as a proxy for assessing hPSC-CM maturity. By integrating biological domain knowledge with machine learning algorithms, we identified key cardiac genes that correlate strongly with culture time. Regression models further predicted culture time with an average error of less than 4.5 days. This work establishes a foundation for standardizing the quantification of hPSC-CM maturation and offers valuable insights for promoting their maturation. Building on this foundation, our future work will focus on developing an advanced lab-on-chip device capable of real-time monitoring of cell maturity and selecting adaptive stimuli to enhance cell maturation.

## Figures and Tables

**Table 1.** The 21 example cardiac-specific genes and fold change expression.

Gene	Description	Adult 1	Adult 2	Day 120	Day 0
*ACTC1*	Actin, alpha, cardiac muscle 1	15.64	15.60	15.48	9.74
*MYH7*	Myosin light chain 7	15.62	15.62	14.02	6.82
*CRYAB*	Crystallin alpha B	15.52	15.48	13.81	6.80
*TNNC1*	Troponin C1, slow skeletal and cardiac type	15.50	15.30	14.57	7.46
*MYL2*	Myosin light chain 2	15.43	15.38	14.56	6.99
*MYL3*	Myosin light chain 3	15.15	15.18	14.60	6.86
*MYH6*	Myosin light chain 6	14.71	15.57	15.01	6.99
*MB*	Myoglobin	14.59	14.50	13.67	6.90
*MYBPC3*	Myosin binding protein C, cardiac	14.54	14.71	13.96	6.85
*TNNT2*	Troponin T2, cardiac type	14.51	14.08	13.72	7.48
*TNNI3*	Troponin I3, cardiac type	14.37	14.06	12.32	7.95
*CKMT2*	Creatine kinase, mitochondrial 2	14.22	14.29	12.45	7.16
*NPPA*	Natriuretic peptide A	14.17	14.95	15.22	6.84
*CASQ2*	Calsequestrin 2	14.08	13.69	12.41	6.92
*HRC*	Histidine rich calcium binding protein	14.02	13.96	11.81	7.38
*MYL7*	Myoslin light chain 7	13.65	14.24	15.07	7.11
*ACTN2*	Actinin alpha 2	12.15	11.31	10.59	7.48
*NKX2-5*	NK2 homeobox 5	11.10	11.03	10.71	6.76
*PLN*	Phospholamban	10.79	8.28	11.50	6.88
*LDB3*	LIM domain binding 3	9.15	8.76	8.92	6.86
*KCNH2*	Potassium voltage-gated channel subfamily H member 2	8.16	8.52	8.07	7.10

**Table 2.** The coefficient of determination.

Time t	${\text{R}}_{{ϒ}_{\text{t}},{ϒ}^{*}}^{2}$	Time t	${\text{R}}_{{ϒ}_{\text{t}},{ϒ}^{*}}^{2}$
Day 0	0.08	Day 28	0.55
Day 3	0.09	Day 35	0.58
Day 7	0.12	Day 45	0.59
Day 10	0.23	Day 60	0.61
Day 14	0.37	Day 90	0.61
Day 20	0.49	Day 120	0.65

**Table 3.** Training and testing datasets.

Dataset	Number of Records	Percentage
Training	$M_{train}=40$	85%
Testing	$M_{test}=8$	15%

**Table 4.** The performance of various feature selection and regression algorithms.

Method	${\text{k}}^{*}$	Genes in ${\text{X}}^{\prime }$ Ranked by Importance	RMSE (Day)	${\text{R}}^{2}$Score
**M**_1_ (Linear)	2	$\left\{x_{19},x_{175}\right\}$	6.837	0.934
**M**_2_ (Linear)	39	$\left\{x_{45},x_{9},x_{159},x_{149},x_{84},x_{74},x_{57},x_{19},x_{28},x_{169},x_{44},x_{60},x_{30},x_{69},\;x_{158},x_{175},x_{94},x_{174},x_{115},x_{3},x_{176},x_{16},x_{133},x_{1},x_{43},x_{83},x_{86},x_{162},x_{91},x_{183},x_{144},x_{14},x_{33},x_{126},x_{15},x_{141},x_{85},x_{157},x_{26}\right\}$	5.216	0.962
**M**_3_ (Linear)	40	$\left\{x_{19},x_{159},x_{176},x_{16},x_{144},x_{149},x_{60},x_{69},x_{183},x_{31},x_{115},x_{86},x_{15},x_{74},x_{1},x_{14},x_{43},x_{33},x_{152},x_{175},x_{38},x_{127},x_{48},x_{94},x_{44},x_{56},x_{162},x_{8},x_{78},x_{167},x_{57},x_{133},x_{85},x_{84},x_{109},x_{23},x_{126},x_{7},x_{142},x_{3}\right\}$	5.521	0.957
**M**_4_ (XGBoost)	27	$\left\{x_{19},x_{93},x_{149},x_{168},x_{123},x_{17},x_{151},x_{144},x_{46},x_{2},x_{82},x_{109},x_{60},x_{4},x_{91},x_{52},x_{159},x_{175},x_{44},x_{49},x_{16},x_{14},x_{94},x_{11},x_{130},x_{7},x_{43}\right\}$	4.461	0.972
**M**_5_ (Linear)	27	$\left\{x_{159},x_{94},x_{168},x_{16},x_{4},x_{91},x_{109},x_{151},x_{82},x_{52},x_{2},x_{17},x_{130},x_{175},x_{11},x_{60},x_{93},x_{19},x_{14},x_{43},x_{149},x_{49},x_{144},x_{123},x_{7},x_{46},x_{44}\right\}$	8.724	0.892

## Data Availability

Data available in a publicly accessible repository: The data presented in this study are openly available in Supp_table4, GSM873339-GSM873374 at 10.1089/scd.2011.0357, reference number [16,25].

## Appendix A

**Table A1.** The mapping between variable name in X and cardiac gene ID.

Notation	Gene	Notation	Gene	Notation	Gene	Notation	Gene
$x_{1}$	*ABO*	$x_{2}$	*ACO2*	$x_{3}$	*ACOT2*	$x_{4}$	*ACOX2*
$x_{5}$	*ACTN2*	$x_{6}$	*AK1*	$x_{7}$	*ANK1*	$x_{8}$	*ANKRD2*
$x_{9}$	*ATP5G1*	$x_{10}$	*ATP5G3*	$x_{11}$	*BAG3*	$x_{12}$	*BRP44L*
$x_{13}$	*BSG*	$x_{14}$	*C1QA*	$x_{15}$	*C1QB*	$x_{16}$	*CA4*
$x_{17}$	*CABC1*	$x_{18}$	*CAND2*	$x_{19}$	*CASQ2*	$x_{20}$	*CCL15*
$x_{21}$	*CD151*	$x_{22}$	*CD320*	$x_{23}$	*CFD*	$x_{24}$	*CHST7*
$x_{25}$	*CKM*	$x_{26}$	*CKMT2*	$x_{27}$	*CLEC3B*	$x_{28}$	*CLTB*
$x_{29}$	*COQ9*	$x_{30}$	*COX5A*	$x_{31}$	*COX5B*	$x_{32}$	*COX6A2*
$x_{33}$	*COX7A1*	$x_{34}$	*COX8A*	$x_{35}$	*CPT1B*	$x_{36}$	*CRIP2*
$x_{37}$	*CRYAB*	$x_{38}$	*CRYM*	$x_{39}$	*CSDC2*	$x_{40}$	*CSRP3*
$x_{41}$	*CTTN*	$x_{42}$	*CYC1*	$x_{43}$	*DCHS1*	$x_{44}$	*DCI*
$x_{45}$	*DES*	$x_{46}$	*DEXI*	$x_{47}$	*DMPK*	$x_{48}$	*DSPP*
$x_{49}$	*ECHDC3*	$x_{50}$	*ECSIT*	$x_{51}$	*EEF1A2*	$x_{52}$	*EFEMP2*
$x_{53}$	*ENDOG*	$x_{54}$	*ERCC1*	$x_{55}$	*FABP3*	$x_{56}$	*FAHD2A*
$x_{57}$	*FARS2*	$x_{58}$	*FHL2*	$x_{59}$	*FLJ22222*	$x_{60}$	*FLNC*
$x_{61}$	*FXYD1*	$x_{62}$	*GADD45GIP1*	$x_{63}$	*GAMT*	$x_{64}$	*GATA4*
$x_{65}$	*GATA6*	$x_{66}$	*GOT1*	$x_{67}$	*GPC1*	$x_{68}$	*GYS1*
$x_{69}$	*HOMER3*	$x_{70}$	*HRC*	$x_{71}$	*HSPB1*	$x_{72}$	*HSPB2*
$x_{73}$	*HSPB3*	$x_{74}$	*HSPB6*	$x_{75}$	*HSPB7*	$x_{76}$	*HSPB8*
$x_{77}$	*HSPG2*	$x_{78}$	*ICAM2*	$x_{79}$	*IDH2*	$x_{80}$	*IFI27*
$x_{81}$	*IGFBP2*	$x_{82}$	*IGFBP7*	$x_{83}$	*IL11RA*	$x_{84}$	*ILVBL*
$x_{85}$	*INMT*	$x_{86}$	*ITGA7*	$x_{87}$	*ITGB1BP2*	$x_{88}$	*ITGB1BP3*
$x_{89}$	*KCNH2*	$x_{90}$	*LDB3*	$x_{91}$	*LGALS3BP*	$x_{92}$	*MAPKAPK3*
$x_{93}$	*MB*	$x_{94}$	*MCOLN1*	$x_{95}$	*MRAS*	$x_{96}$	*MRPL12*
$x_{97}$	*MRPL34*	$x_{98}$	*MRPL41*	$x_{99}$	*MRPS12*	$x_{100}$	*MSRB2*
$x_{101}$	*MYBPC3*	$x_{102}$	*MYH6*	$x_{103}$	*MYH7*	$x_{104}$	*MYL2*
$x_{105}$	*MYL3*	$x_{106}$	*MYL4*	$x_{107}$	*MYL7*	$x_{108}$	*MYL9*
$x_{109}$	*MYLC2PL*	$x_{110}$	*MYOM1*	$x_{111}$	*MYOM2*	$x_{112}$	*MYOZ2*
$x_{113}$	*NDUFA11*	$x_{114}$	*NDUFA7*	$x_{115}$	*NDUFB10*	$x_{116}$	*NDUFB7*
$x_{117}$	*NDUFS7*	$x_{118}$	*NDUFS8*	$x_{119}$	*NKX2-5*	$x_{120}$	*NOL3*
$x_{121}$	*NPPA*	$x_{122}$	*NPPB*	$x_{123}$	*NRAP*	$x_{124}$	*OGDH*
$x_{125}$	*OPLAH*	$x_{126}$	*PCTK3*	$x_{127}$	*PDE4DIP*	$x_{128}$	*PDK2*
$x_{129}$	*PDLIM5*	$x_{130}$	*PGAM2*	$x_{131}$	*PGM1*	$x_{132}$	*PHPT1*
$x_{133}$	*PLA2G5*	$x_{134}$	*PLEKHF1*	$x_{135}$	*PLN*	$x_{136}$	*POLR2I*
$x_{137}$	*POLRMT*	$x_{138}$	*POMGNT1*	$x_{139}$	*POPDC2*	$x_{140}$	*PPAPDC3*
$x_{141}$	*PPP1R13L*	$x_{142}$	*PPP1R1A*	$x_{143}$	*PPP2R3B*	$x_{144}$	*PTGDS*
$x_{145}$	*PTP4A3*	$x_{146}$	*PTPLA*	$x_{147}$	*PTRF*	$x_{148}$	*PXMP2*
$x_{149}$	*RAMP1*	$x_{150}$	*RAMP3*	$x_{151}$	*RASIP1*	$x_{152}$	*RBPMS*
$x_{153}$	*RGS3*	$x_{154}$	*RRAS*	$x_{155}$	*S100A1*	$x_{156}$	*SEPW1*
$x_{157}$	*SGCG*	$x_{158}$	*SH3RF2*	$x_{159}$	*SIVA*	$x_{160}$	*SLC25A11*
$x_{161}$	*SLC25A4*	$x_{162}$	*SLC29A1*	$x_{163}$	*SLC4A3*	$x_{164}$	*SMPX*
$x_{165}$	*SMTN*	$x_{166}$	*SNTA1*	$x_{167}$	*STAB1*	$x_{168}$	*STOML1*
$x_{169}$	*STOML2*	$x_{170}$	*SYNPO2L*	$x_{171}$	*TACC2*	$x_{172}$	*TAX1BP3*
$x_{173}$	*TCAP*	$x_{174}$	*TIMM8B*	$x_{175}$	*TM7SF2*	$x_{176}$	*TMEM159*
$x_{177}$	*TNNC1*	$x_{178}$	*TNNI3*	$x_{179}$	*TNNT2*	$x_{180}$	*TNXA*
$x_{181}$	*TNXB*	$x_{182}$	*TPM1*	$x_{183}$	*TSPAN4*	$x_{184}$	*UQCR*
$x_{185}$	*UQCRC1*	$x_{186}$	*VAMP5*	$x_{187}$	*VEGFB*	$x_{188}$	*VWF*
$x_{189}$	*WDR13*						
